# Supplementary material for: A single-cell and spatial RNA-seq database for Alzheimer’s disease (ssREAD)
Source: Nat Commun. 2024 Jun 6;15:4710. doi: 10.1038/s41467-024-49133-z (PMC11156951; doi:10.1038/s41467-024-49133-z)
Supplement: Supplementary file 1 — Supplementary Information [file 41467_2024_49133_MOESM1_ESM.pdf]

Supplementary Information of
A Single-cell and Spatial RNA-seq Database for Alzheimer's Disease (ssREAD)

Cankun Wang[1], Diana Acosta[2], Megan McNutt[1], Jiang Bian[3], Anjun Ma[1], Hongjun Fu[2,4*], Qin Ma[1,*]

[1] Department of Biomedical Informatics, The Ohio State University, OH 43210, USA

[2] Department of Neuroscience, The Ohio State University, OH 43210, USA

[3] Department of Health Outcomes & Biomedical Informatics, University of Florida, FL 32606, USA

[4] Chronic Brain Injury Program, The Ohio State University, OH 43210, USA

*Correspondence: hongjun.fu@osumc.edu and qin.ma@osumc.edu

Tutorial of ssREAD

1. **ssREAD webpage introduction**

- Home
    - Flow diagram of available spatial and scRNA-seq datasets.
    - Bar graph of datasets available from each brain region.
    - Pie cart of available study types and conditions.
    - Interactive plot for visualizing cell types and spatial layers.
- Browse
    - scRNA-seq datasets: 277 datasets from 1,053 samples, 67 studies, and 7,332,202 cells.
    - Spatial Transcriptomics datasets: 381 samples from 18 studies.
    - Sort by species, brain region, condition/experimental factors, and sex/gender.
- Query
    - Search differentially expressed genes (DEGs).
    - Find overlapping differentially expressed genes from multiple comparisons from a cell type of interest in a specific brain region and set your own parameters.
- Help
    - Usage
    - Frequently asked questions
    - Contact
    - News: updates on ssREAD
- Download
    - Download raw and processed datasets
    - Steps to run ssREAD backend workflow locally

2. **Example Result Illustration**

We used the dataset ST00109 to showcase the functions of the database. It comes from the dorsolateral prefrontal cortex region of a 30-year-old, Caucasian female in a non-diseased state in Maynard, et al. research. This tutorial will guide you through the analysis result page of ssREAD in detail.

2.1 General Information

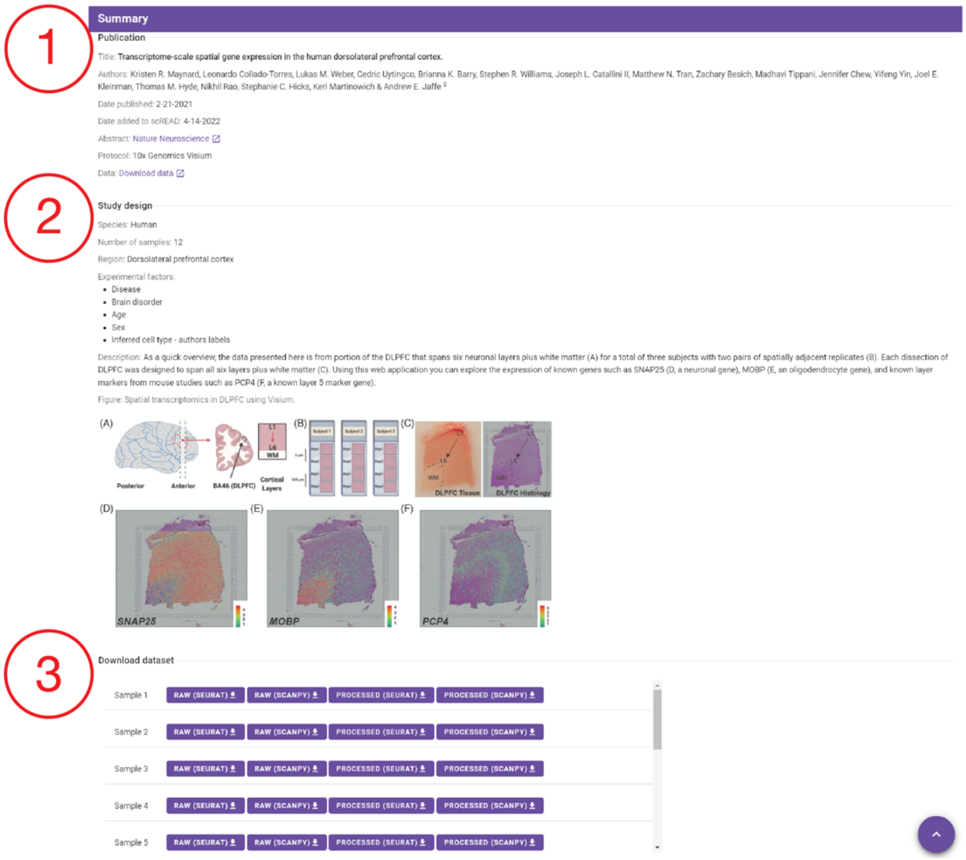

The summary of this dataset is shown in the first section of the analysis result page. This section includes three parts: (1) Publication, (2) Study design, and (3) Download dataset.

1. Publication Overview: 'Title', 'Date published', 'Date added to ssREAD', 'Abstract', 'Protocol', and 'Data'.
2. Study Design: 'Species', 'Number of Samples', 'Region', 'Experimental factors', 'Description', and 'Figure: Spatial transcriptomics in DLPFC using Visium'.
3. Download dataset provides the link to download the raw or processed data via Seurat or Scanpy.

2.2 Spot-level data

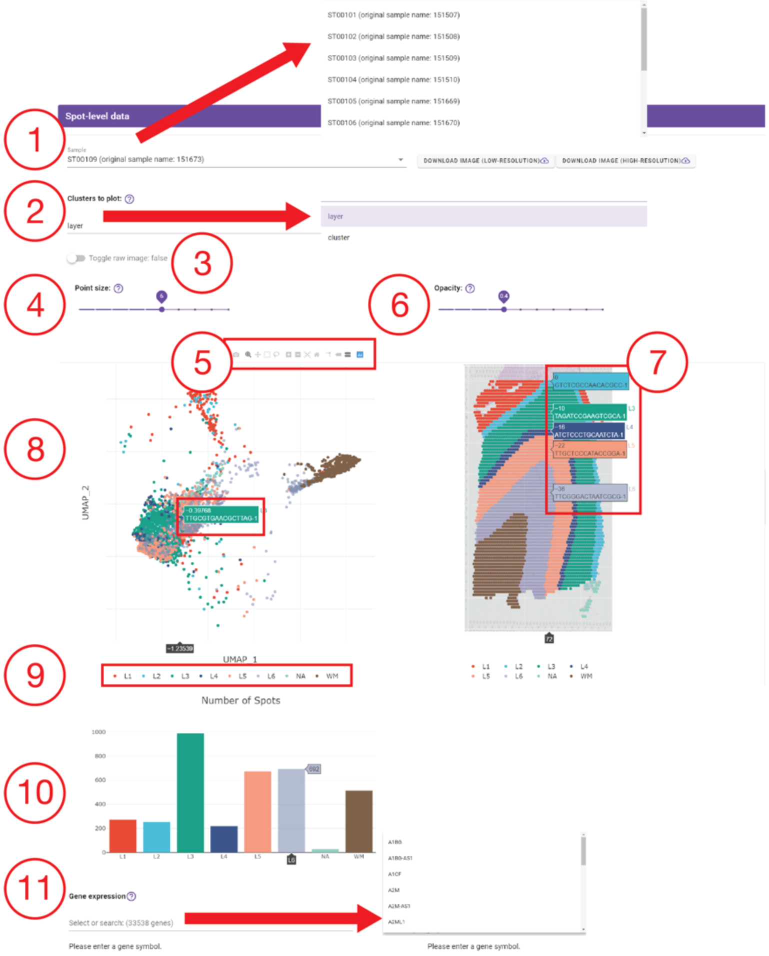

The 'Spot-level data' section allows users to select specific samples from the dataset and manipulate interactive UMAP plots that show predicted cell types, the expression and distribution of genes, and the number of spots of each cell type.

1. Users select their desired sample from the dataset. For this example, ST00109 was used. The following UMAP will change to the UMAP of predicted subclusters for this specific sample.
2. Choose to plot by layer or cluster.
3. View the processed or raw image.
4. A sliding bar is used to control the size of each point in the UMAP. It ranges from 1 (smallest) to 10 (largest).
5. This function bar contains several quick buttons for graphic operations.
6. A sliding bar is used to control the opacity of the underlying raw image. It ranges from 1 (translucent) to 10 (completely opaque).
7. Hover the cursor on cell points to display cell type, cell name, and UMAP coordinates.

8. Hover the cursor on cell points to display cell type, cell name, and UMAP coordinates.
9. The legend of this UMAP plot.
10. Hover the cursor on a bar to display the number of spots and cell type.
11. Select or search a gene of interest to plot the gene expression value on the UMAP plot. A darker point will indicate a higher expression value.

2.3 Spatially variable genes

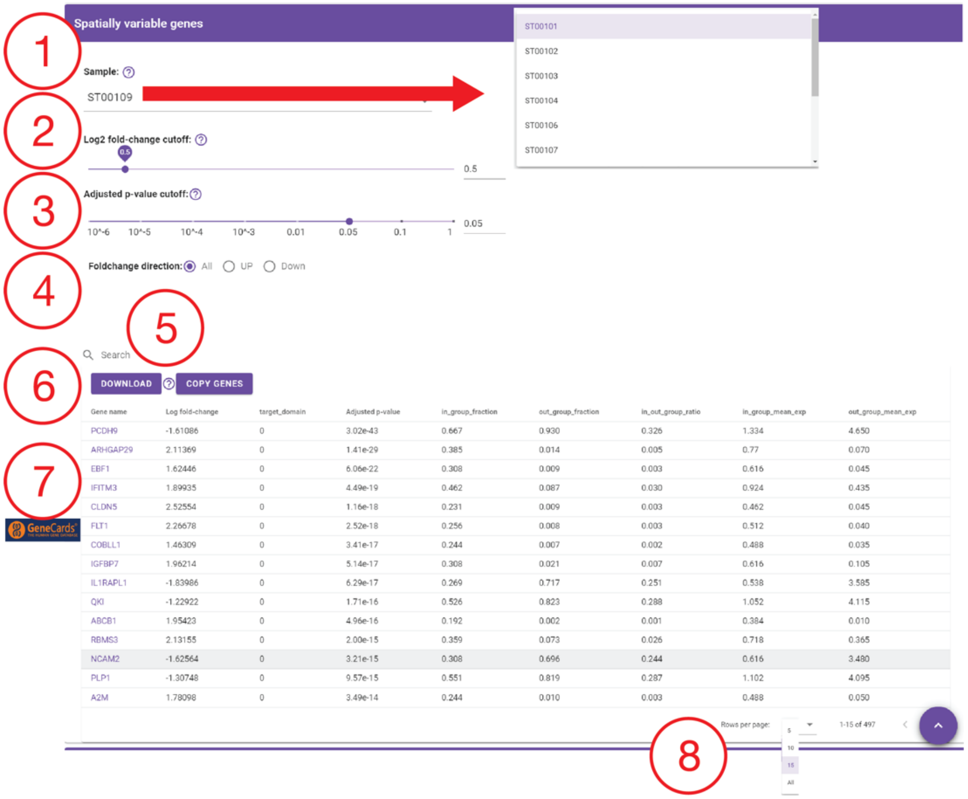

1. Users select their desired sample from the dataset. For this example, ST00109 was used.
2. A sliding bar is used to change the Log2 fold-change cutoff. This ranges from 0 to 5.
3. A sliding bar is used to vary the adjusted p-value from 10^-6 to 1.
4. The foldchange direction can be filtered by all spatially variable genes, only upregulated genes, or only downregulated genes.
5. Users can search for genes that they are interested in, and then the following table will return the matching result.
6. Download or copy the currently listed table.
7. Genecards database is linked to each gene in the table.
8. Set how many rows the table shows.

2.4 Differential expression (DE)/ Gene set enrichment

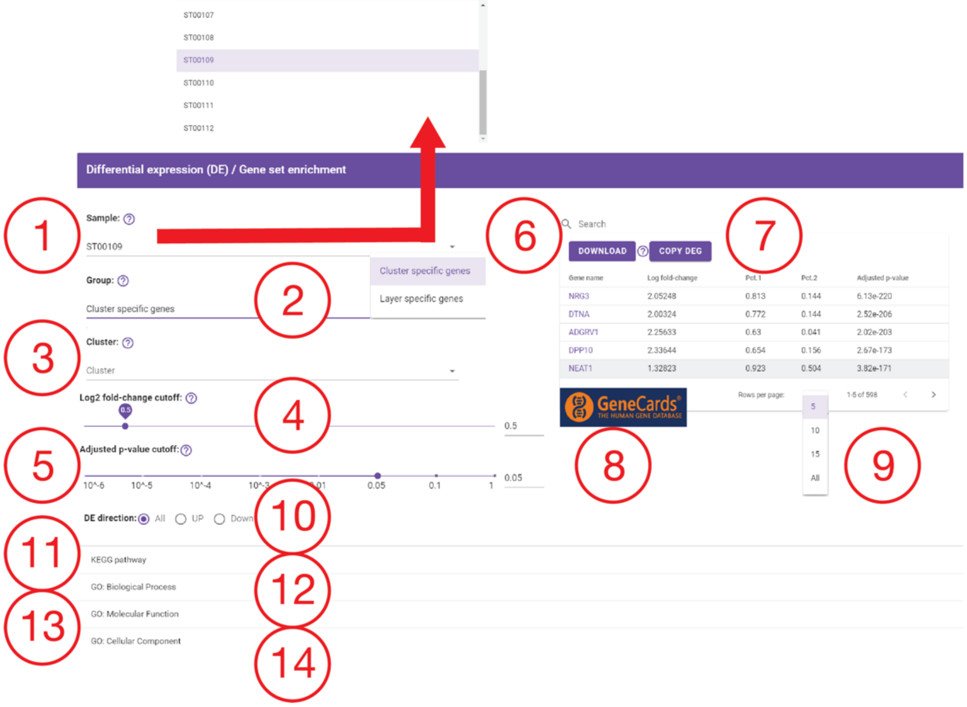

1. Users select their desired sample from the dataset. For this example, ST00109 was used.
2. Users select to either cluster specific genes or layer specific genes between two sets of cells.
3. Users select the cell type to perform differential analysis.

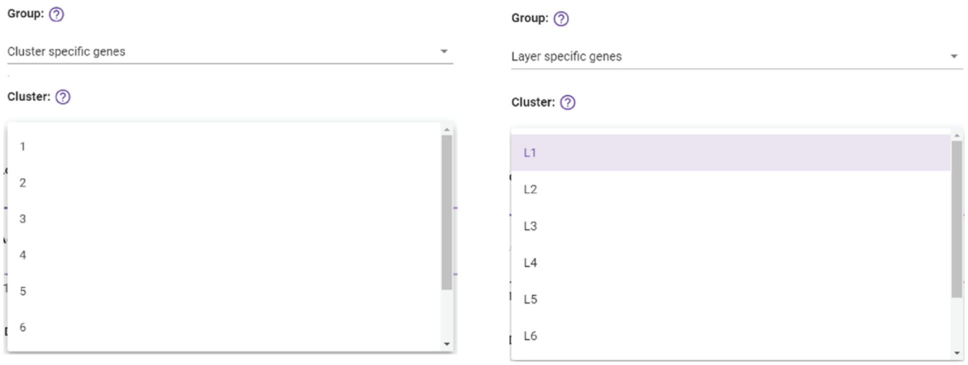

4. The Log2 fold-change ranges from 0 to 5.
5. The Adjusted p-value ranges from 10^-6 to 1.
6. Users can search for genes that they are interested in, and the table will return matching results.
7. Users can download or copy the table.
8. Genecards database is linked to each gene in the table.
9. Users can set how many rows are shown in the table.

10. The DE direction can be filtered by all DE genes, only upregulated genes, or only downregulated genes.

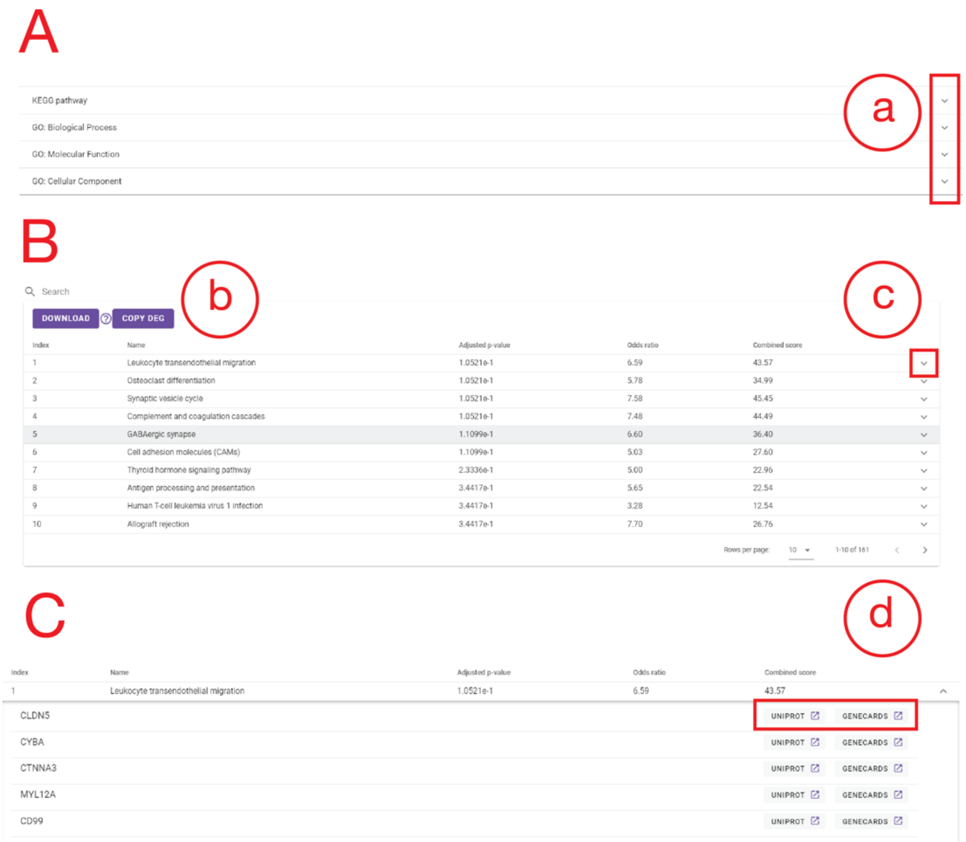

11. KEGG pathway enrichment analysis result table B of the DEGs will appear when the user clicks on the drop-down arrow (a). This table can be downloaded or copied when the user clicks on the 'Download' or 'Copy DEG' button (b). If the user clicks on the drop-down arrow (c) in table B, they can see the genes that are enriched on this pathway. This table can also be copied or downloaded via the 'Download' or 'Copy DEG' functions. Users can click on the links to UNIPROT or GENECARDS to view the results for each gene in these databases (d).
    A. GO Biological Process analysis result of the DEGs. Entry 11 above can be used to navigate this feature.
    B. GO Molecular Function analysis result of the DEGs. Entry 11 above can be used to navigate this feature.
    C. GO Cellular Component analysis result of the DEGs. Entry 11 above can be used to navigate this feature.

2.5 Deconvolution

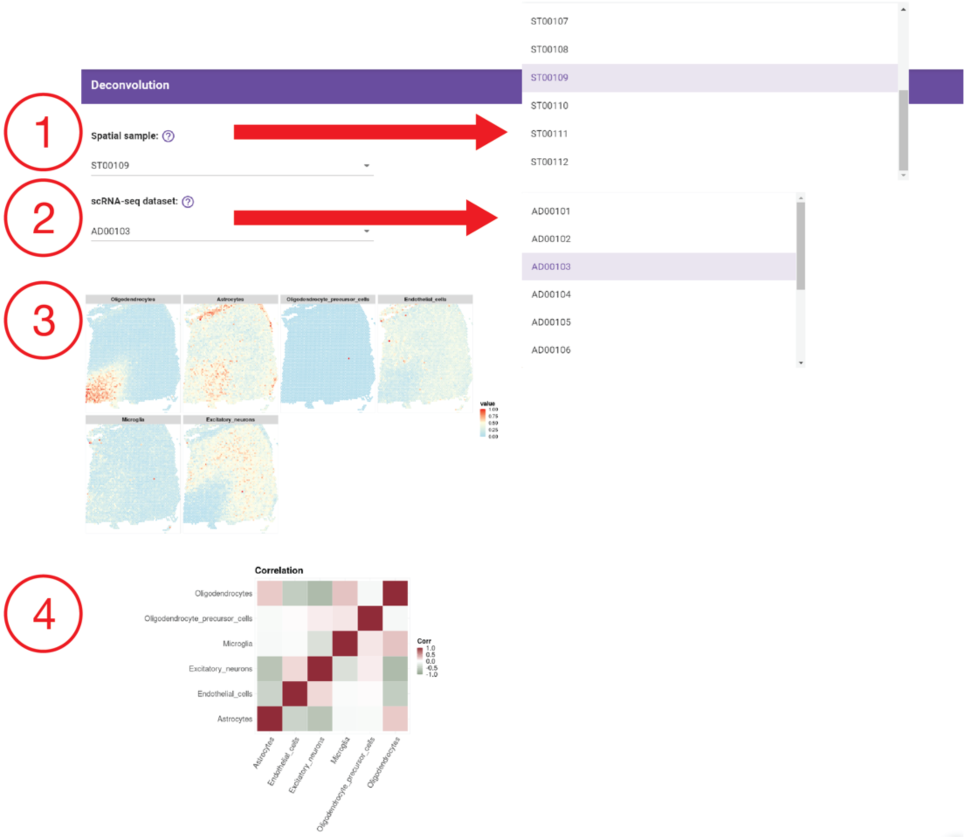

1. Users select desired spatial transcriptomics sample from the dataset. For this example, ST00109 was used.
2. Users select desired scRNA-seq dataset to compare to the spatial sample and perform cell-type deconvolution.
3. Average gene expression (scaled 0-1) for each spot from a mixture of cells of potentially heterogeneous cell types. The outputs are the estimated cell-type composition across spatial locations for the corresponding scRNA-seq dataset.
4. Correlations in proportions of cell-types across spatial locations between cell-type pairs, where correlations range from -1 (dark sage green) to 1 (burgundy).

3. ssREAD Browse function

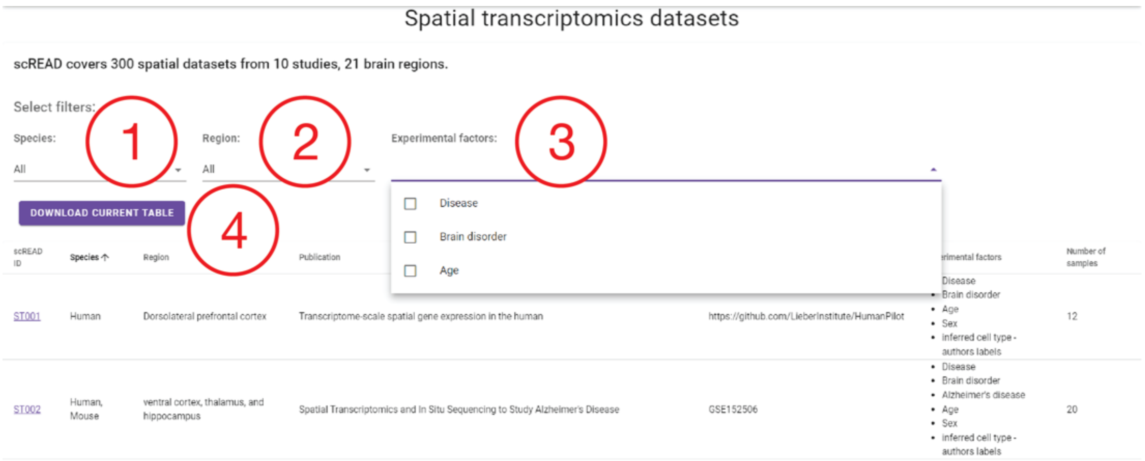

The 'Browse' page allows users to browse scRNA-seq or spatial transcriptomics datasets that are stored in ssREAD based on factors such as species, brain region, or experimental factors.

1. Users can sort by species (mouse or human) via the drop-down arrow.
2. Users can sort by brain region by via the drop-down arrow.
3. Users can sort by experimental factors such as disease, brain disorder, or age by via the drop-down arrow.
4. Users can download the table by via 'Download Current Table'.

4. ssREAD Query function

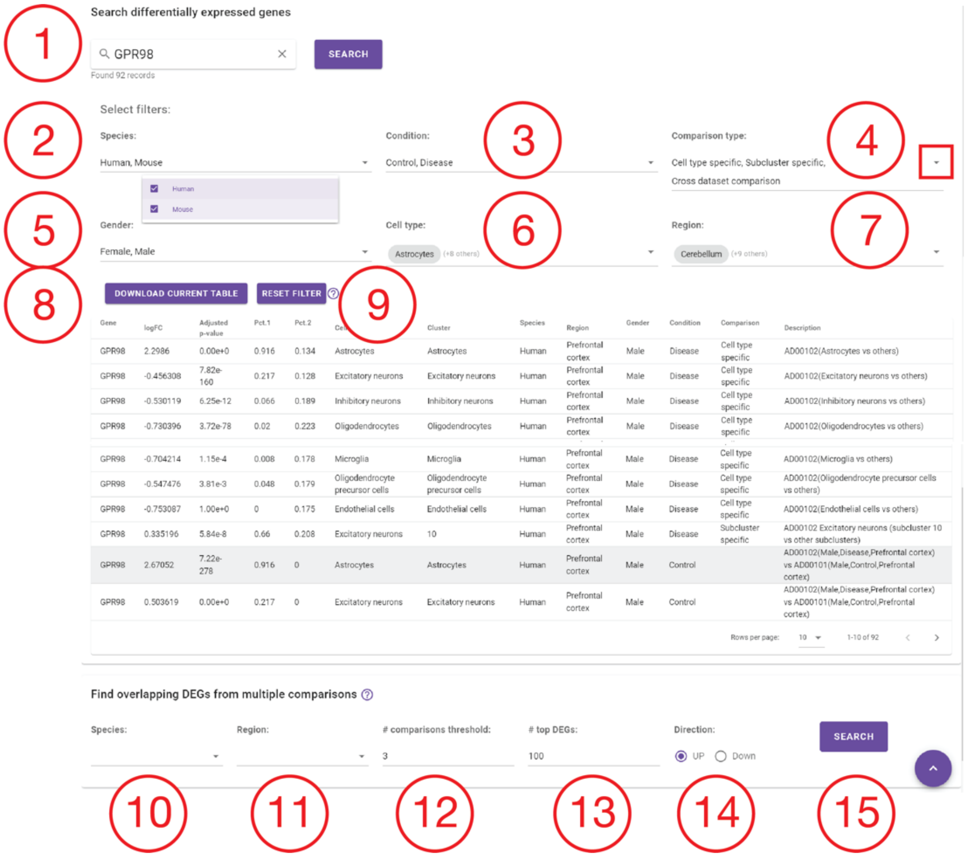

The 'Query' page allows users to search for specific DEGs by using different filters. Users can also find overlapping DEGs from multiple comparisons.

1. Users can search for a differentially expressed gene of their choice. For this example, GPR98 was used.
2. Users can select the drop-down arrow to filter by species: human or mouse.

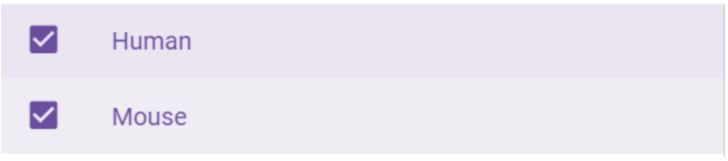

3. Users can select the drop-down arrow to filter by experimental condition: control or disease.

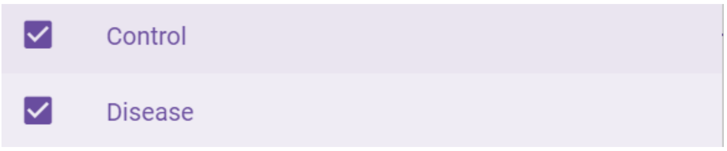

4. Users can select the drop-down arrow to filter by comparison type: cell type specific, subcluster specific, or cross dataset comparison.

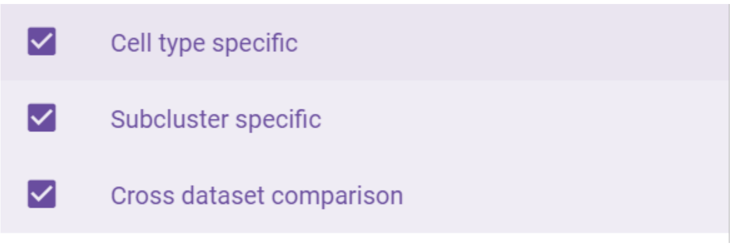

5. Users can select the drop-down arrow to filter by subject sex.

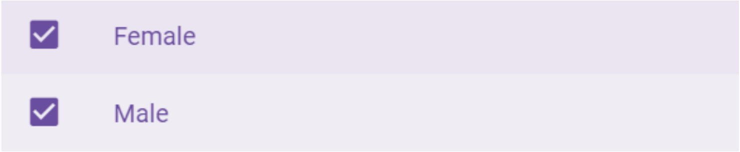

6. Users can select the drop-down arrow to filter by cell type.

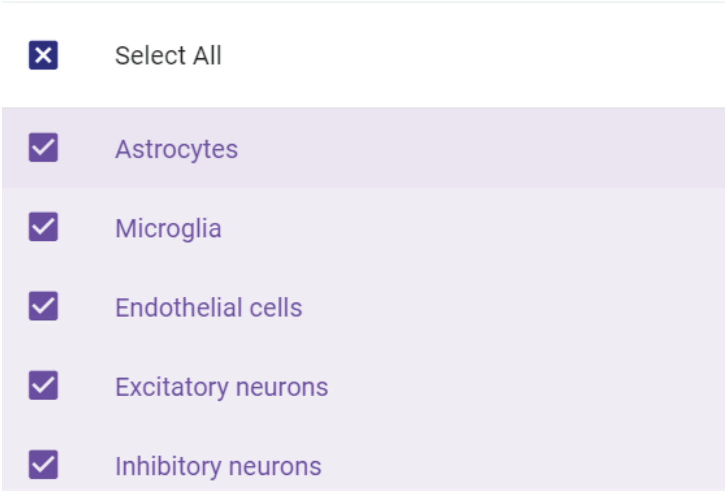

7. Users can select the drop-down arrow to filter by brain region.

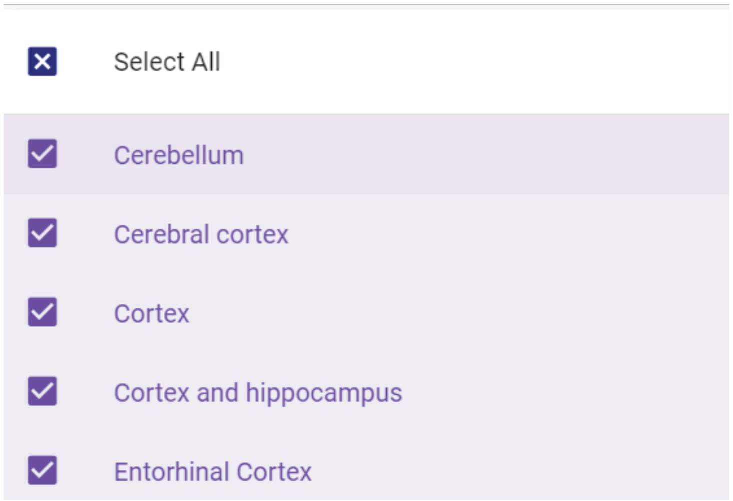

8. Users can select the 'Download Current Table' button to download the produced table.

9. Users can click on the 'Reset Filter' button to erase their selected filters and return to default.
10. Users can select from the drop-down menu what species (mouse or human) they want to search for when finding overlapping DEGs from multiple comparisons.
11. Users can select from the drop-down menu what brain region they want to search for when finding overlapping DEGs from multiple comparisons.
12. Users can input the number of comparisons threshold for their overlapping DEGs search.
13. Users can input the number of top DEGs they want to see in their overlapping DEGs search.
14. The DE direction can be filtered by all DE genes, only upregulated genes, or only downregulated genes.
15. Click on the 'Search' button to produce results of overlapping DEGs from multiple comparisons.

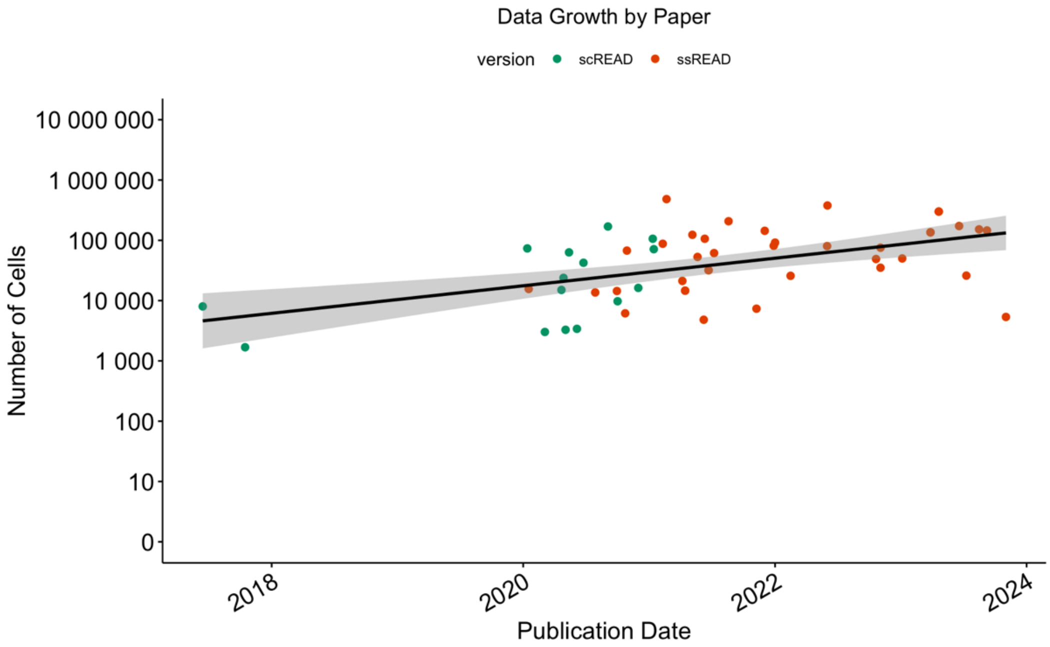

Supplementary Fig. 1. Yearly growth in the number of cells collected from sc/snRNA-seq publications by scREAD and ssREAD. The scatter plot displays the cell count on a logarithmic scale (y-axis) against the publication year (x-axis).

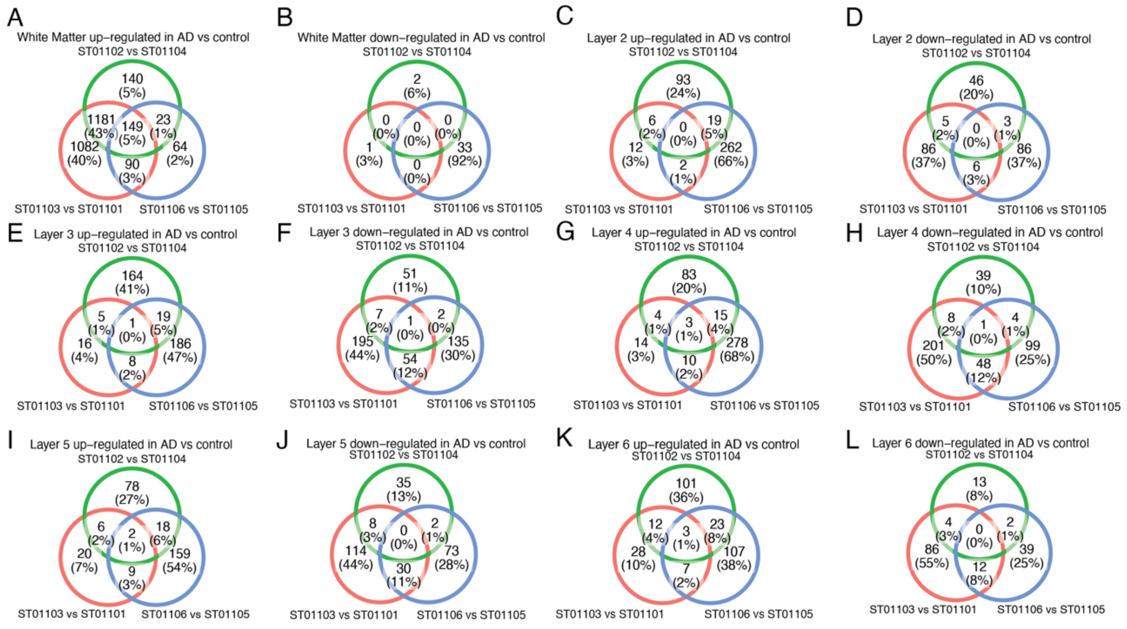

Supplementary Fig. 2. Overlap of DEGs in Human MTG integrated spatial datasets. Venn diagrams depict the shared DEGs across comparisons within dataset ID ST011. Panels show the up and downregulated DEGs for each specific brain layer in the following comparisons: AD01103 vs. ST01101, ST01102 vs. ST01104, and ST01106 vs. ST01105. (A, B) White Matter, (C, D) Layer 2, (E, F) Layer 3, (G, H) Layer 4, (I, J) Layer 5, (K, L) Layer 6.

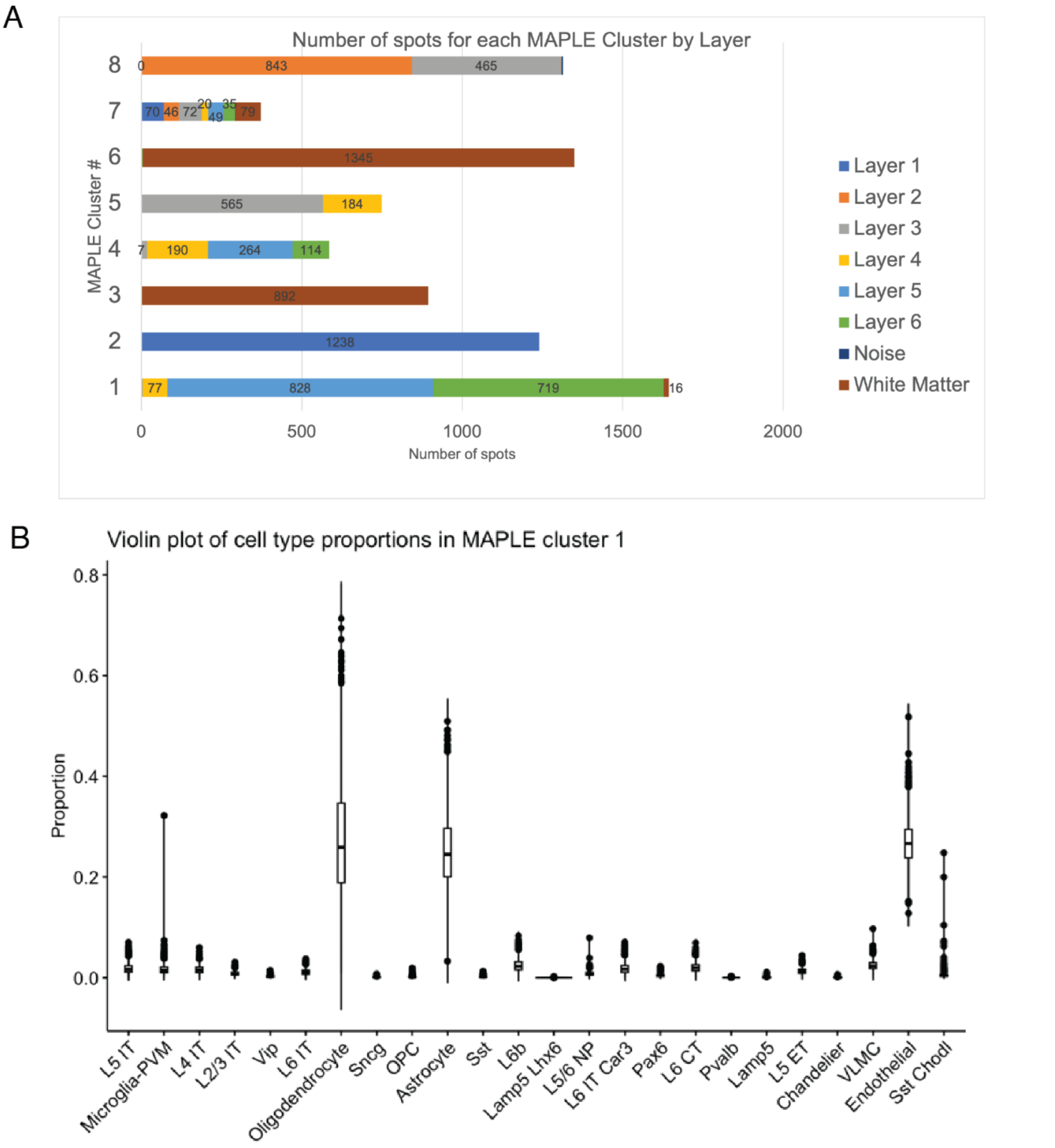

Supplementary Fig. 3. (**A**) Bar plot displays the distribution of spots by cluster and layer corresponding to MAPLE clusters in datasets ST01301 and ST01303. The data indicates that most cells are found in layers 5/6 in MAPLE cluster 1. (**B**) Cell type proportions in MAPLE cluster 1 were determined by deconvolution analysis. The violin plot visually represents the distribution of cell types in MAPLE cluster 1. Notably, cluster 1 predominantly consists of Astrocytes, Oligodendrocytes, and Endothelial cells.

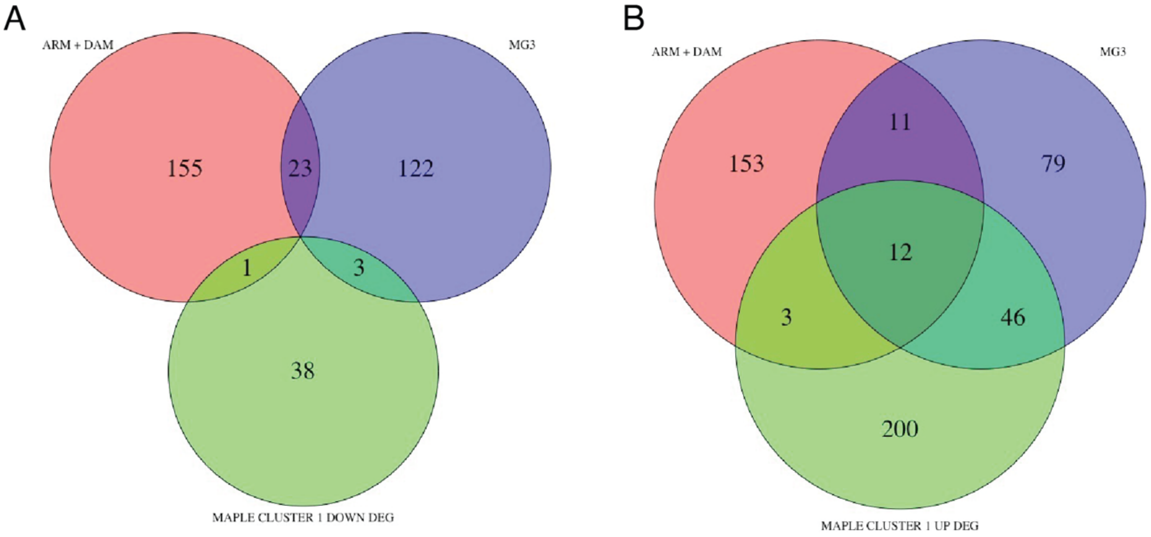

Supplementary Fig. 4. Venn Diagram of genes associated with each pathway identified within datasets ST01301 and ST01303 MAPLE Cluster 1, as well as their overlap with genes corresponding to disease-associated microglia states. No overlap is observed in panel (**A**), which indicates genes associated with downregulated pathways are not found in disease-associated microglia gene markers, while a larger overlap is observed in panel (B), which indicates genes within upregulated pathways are also considered disease-associated microglia gene markers. ARM: activated response microglia; DAM: disease-associated microglia; MG3: a population of microglia that is highly enriched with disease-associated microglial genes from Sun *et al*., Cell 186.20 (2023): 4386-4403.

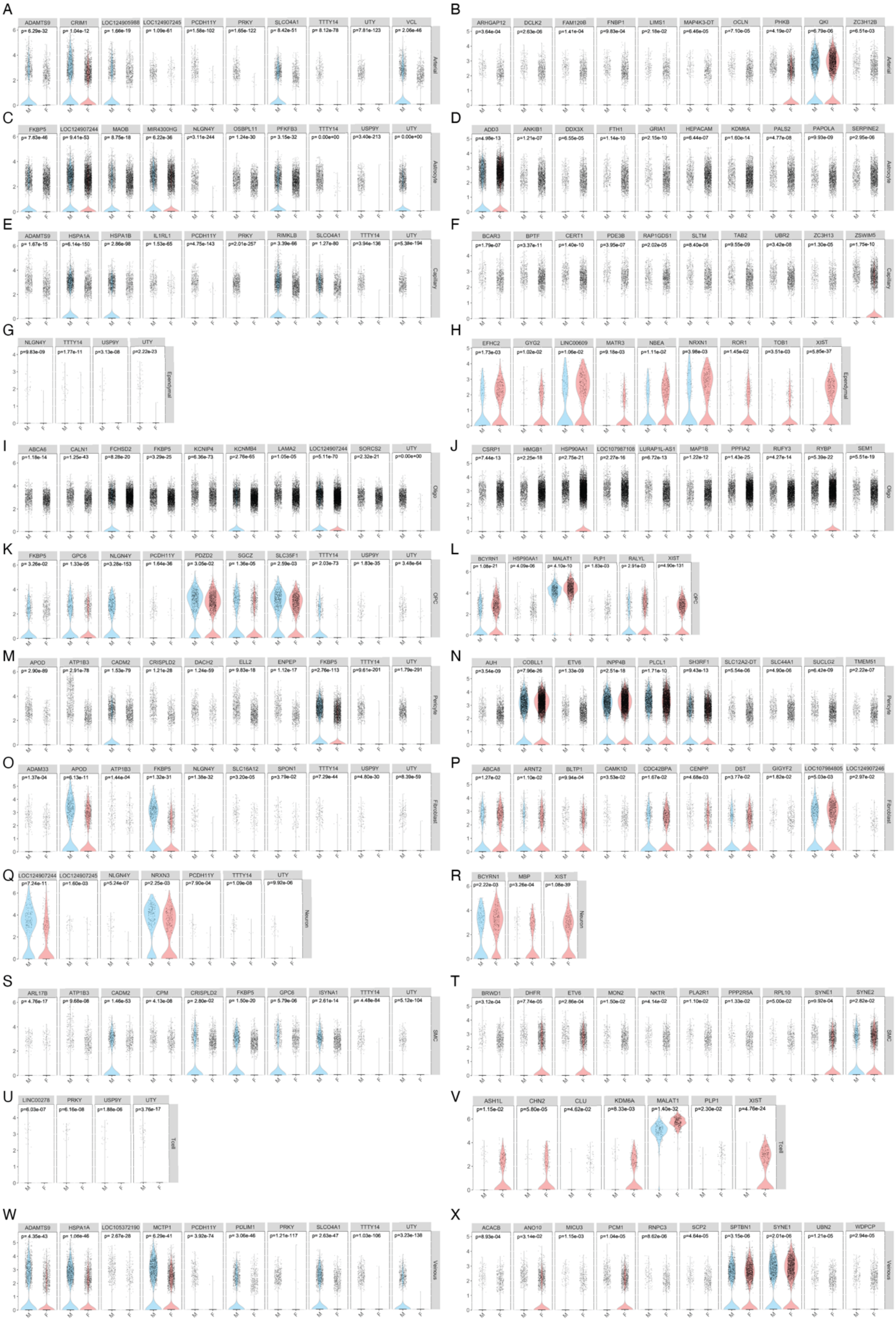
A
B
C
D
E
F
G
H
I
J
K
L
M
N
O
P
Q
R
S
T
U
V
W
X

Supplementary Fig. 5. Violin plots from the integrated AD019 datasets representing the top 10 upregulated (left) and top 10 downregulated (right) differentially expressed genes (DEGs) in males versus females, spanning across 14 distinct cell types. *p*-values were calculated based on a two-sided Wilcoxon Rank-Sum test and adjusted using Bonferroni correction. The cell types analyzed are as follows: Arterial (A-B), Astrocyte (C-D), Capillary (E-F), Ependymal (G-H), Fibroblast (I-J), Neuron (K-L), Oligodendrocytes (M-N), Oligodendrocyte Precursor Cells (OPC) (O-P), Pericyte (Q-R), Smooth Muscle Cells (SMC) (S-T), T cells (U-V), and Venous (W-X). It is important to note that not all cell types yielded a full complement of the top 10 DEGs; as such, some violin plots may depict fewer genes.
