# Supplementary material for: A single-cell and spatial RNA-seq database for Alzheimer’s disease (ssREAD)
Source: Nat Commun. 2024 Jun 6;15:4710. doi: 10.1038/s41467-024-49133-z (PMC11156951; doi:10.1038/s41467-024-49133-z)
Supplement: Supplementary file 3 — Description of Additional Supplementary Files [file 41467_2024_49133_MOESM3_ESM.pdf]

File Name: **Supplementary Data 1**
Description: **All data resources for single-cell RNA-seq and spatial transcriptomics data in ssREAD database.**

File Name: **Supplementary Data 2**
Description: **DEGs between samples ST01103 and ST01101 in white matter and layers 2-6.**

File Name: **Supplementary Data 3**
Description: **DEGs between samples ST01102 and ST01104 in white matter and layers 1-6.**

File Name: **Supplementary Data 4**
Description: **DEGs between samples ST01106 and ST01105 in white matter and layers 1-6.**

File Name: **Supplementary Data 5**
Description: **DEGs identified between ST01103 AD and ST01101 control samples in MAPLE cluster 1-8 comparing, respectively.**

File Name: **Supplementary Data 6**
Description: **TF regulons identified in MAPLE cluster 1 in ST01101 and ST01103.**

File Name: **Supplementary Data 7**
Description: **ATF6 and EGR1 regulon-enriched pathways related to stress response from MAPLE cluster 1 in AD01103 and AD01101.**

File Name: **Supplementary Data 8**
Description: **DEGs identified between AD and control samples in the AD035 MTG dataset in Astrocytes, Endothelial, Excitatory neurons, Inhibitory neurons, Microglia, Oligodendrocytes, and Oligodendrocyte precursor cells, respectively.**

File Name: **Supplementary Data 9**
Description: **DEGs identified between AD and control samples in the AD048 PFC dataset in Astrocytes, Endothelial, Excitatory neurons, Inhibitory neurons, Microglia, Oligodendrocytes, and Oligodendrocyte precursor cells, respectively.**

File Name: **Supplementary Data 10**
Description: **DEGs between AD and control samples in the AD035 MTG and AD048 PFC integrated dataset in Astrocytes, Endothelial, Excitatory neurons, Inhibitory neurons, Microglia, Oligodendrocytes, and Oligodendrocyte precursor cells,**

respectively.

File Name: **Supplementary Data 11**
Description: **Top 25 DEGs identified in integrated AD035 MTG and AD048 PFC datasets alongside comparisons with previously published datasets ST011.**

File Name: **Supplementary Data 12**
Description: **DEGs overlap among AD035 MTG, AD048 PFC, and their integrated datasets, associated with Figure 4H.**

File Name: **Supplementary Data 13**
Description: **DEGs between AD and control datasets in Microglia which overlap with previously published spatial transcriptomics data.**

File Name: **Supplementary Data 14**
Description: **Metadata for sex-specific difference analysis in AD patient sample AD019.**

File Name: **Supplementary Data 15**
Description: **DEGs between male and female AD patients in the integrated AD019 hippocampus dataset in Arterial, Astrocytes, Capillary, Ependymal, Fibroblast, Microglia, Neuron, Oligodendrocytes, Oligodendrocyte precursor cells, Pericyte, Smooth muscle cell, T cell, and Veinous, respectively.**

File Name: **Supplementary Data 16**
Description: **DEGs between male and female AD patients in the integrated AD035 MTG dataset in Astrocytes, Endothelial, Excitatory neurons, Inhibitory neurons, Microglia, Oligodendrocytes, and Oligodendrocyte precursor cells, respectively.**

File Name: **Supplementary Data 17**
Description: **DEGs between male and female AD patients in the integrated AD048 PFC dataset in Astrocytes, Endothelial, Excitatory neurons, Inhibitory neurons, Microglia, Oligodendrocytes, and Oligodendrocyte precursor cells, respectively.**

File Name: **Supplementary Data 18**
Description: **Categorization of brain regions defined in ssREAD.**

File Name: **Supplementary Data 19**
Description: **The marker genes used to assign a cell to a specific cell type.**
