# Supplementary material for: A single-cell and spatial RNA-seq database for Alzheimer’s disease (ssREAD)
Source: Nat Commun. 2024 Jun 6;15:4710. doi: 10.1038/s41467-024-49133-z (PMC11156951; doi:10.1038/s41467-024-49133-z)
Supplement: Supplementary file 23 — Reporting Summary [file 41467_2024_49133_MOESM23_ESM.pdf]

nature portfolio

Corresponding author(s): Hongjun Fu, Qin Ma

Last updated by author(s): Sep 7, 2023

Reporting Summary

Nature Portfolio wishes to improve the reproducibility of the work that we publish. This form provides structure for consistency and transparency in reporting. For further information on Nature Portfolio policies, see our Editorial Policies and the Editorial Policy Checklist.

Statistics

For all statistical analyses, confirm that the following items are present in the figure legend, table legend, main text, or Methods section.

n/a	Confirmed	
☐	☒	The exact sample size (*n*) for each experimental group/condition, given as a discrete number and unit of measurement
☐	☒	A statement on whether measurements were taken from distinct samples or whether the same sample was measured repeatedly
☐	☒	The statistical test(s) used AND whether they are one- or two-sided<br>*Only common tests should be described solely by name; describe more complex techniques in the Methods section.*
☒	☐	A description of all covariates tested
☒	☐	A description of any assumptions or corrections, such as tests of normality and adjustment for multiple comparisons
☐	☒	A full description of the statistical parameters including central tendency (e.g. means) or other basic estimates (e.g. regression coefficient) AND variation (e.g. standard deviation) or associated estimates of uncertainty (e.g. confidence intervals)
☐	☒	For null hypothesis testing, the test statistic (e.g. *F*, *t*, *r*) with confidence intervals, effect sizes, degrees of freedom and *P* value noted<br>*Give P values as exact values whenever suitable.*
☒	☐	For Bayesian analysis, information on the choice of priors and Markov chain Monte Carlo settings
☒	☐	For hierarchical and complex designs, identification of the appropriate level for tests and full reporting of outcomes
☒	☐	Estimates of effect sizes (e.g. Cohen's *d*, Pearson's *r*), indicating how they were calculated

Our web collection on statistics for biologists contains articles on many of the points above.

Software and code

Policy information about availability of computer code

Data collection	Supplemental Information
Data analysis	The frontend code is available at https://github.com/OSU-BMBL/ssread. The backend code is available at https://github.com/OSU-BMBL/ssread-backend. Additionally, a real-time ssREAD server status page is available at https://ssread.statuspage.io/. Other tools and packages used in this paper include: Python v3.8, R v4.3.0, Node.js v20.4, Seurat v5, Squidpy v1.3.0, spaGCN v1.2.7, SCINA v1.2.0, CARD v1.1, MAST v1.28.0, CellChat v2, RESEPT v1, DeepMAPS v1.0.

For manuscripts utilizing custom algorithms or software that are central to the research but not yet described in published literature, software must be made available to editors and reviewers. We strongly encourage code deposition in a community repository (e.g. GitHub). See the Nature Portfolio guidelines for submitting code & software for further information.

Data

Policy information about availability of data

All manuscripts must include a data availability statement. This statement should provide the following information, where applicable:

- Accession codes, unique identifiers, or web links for publicly available datasets
- A description of any restrictions on data availability
- For clinical datasets or third party data, please ensure that the statement adheres to our policy

ssREAD is freely available at https://bmblx.bmi.osumc.edu/ssread/. A backup link is also provided at https://go.osu.edu/ssread. Relevant raw data from each figure

is available in the Source Data file. All datasets used in this manuscript were previously released by other researchers and are publicly available online. The processed data in this study can be downloaded through the link https://bmblx.bmi.osumc.edu/ssread/downloads.

Research involving human participants, their data, or biological material

Policy information about studies with human participants or human data. See also policy information about sex, gender (identity/presentation), and sexual orientation and race, ethnicity and racism.

Reporting on sex and gender	Throughout the manuscript, we have consistently used the term "sex." Our findings are applicable to all genders. Sex-specific information was incorporated in our case study, as depicted in Figure 5. This information was sourced from the original public data, which had been de-identified to protect personal privacy. By leveraging the rich insights provided by sc/snRNA-seq data, we have elucidated the nuanced differences in AD manifestation at the cellular level, influenced by sex.
Reporting on race, ethnicity, or other socially relevant groupings	While ethnicity data is presented in our Figure 4 case study, we have not built our categorizations based on this information. This ethnicity information was originally provided in the public dataset and has been included to offer a comprehensive overview of the data source we utilized.
Population characteristics	In our study, we emphasized the comparison of different Braak pathology stages of AD patients. Braak staging is an established method for defining the extent of neurofibrillary tangle deposition in AD and served as our primary criterion for delineating progression and severity. While age can often be a confounding factor in AD studies, we ensured that our analyses considered its potential effects. Moreover, information regarding past treatments or current diagnoses, where available, was sourced from the original public dataset. These parameters helped us ensure that the variations observed across different Braak stages were not simply attributed to age or treatment differences but were indeed reflective of the distinct pathological states of the disease.
Recruitment	For this study, we did not directly recruit participants. Instead, all data utilized were collected from the public domain. These datasets have been previously published and are available for research purposes.
Ethics oversight	N/A

Note that full information on the approval of the study protocol must also be provided in the manuscript.

Field-specific reporting

Please select the one below that is the best fit for your research. If you are not sure, read the appropriate sections before making your selection.

☒ Life sciences ☐ Behavioural & social sciences ☐ Ecological, evolutionary & environmental sciences

For a reference copy of the document with all sections, see nature.com/documents/nr-reporting-summary-flat.pdf

Life sciences study design

All studies must disclose on these points even when the disclosure is negative.

Sample size	Not applicable
Data exclusions	All cells and genes of each single-cell data set were used, and no exclusion was done prior to analysis.
Replication	We did not perform biological or technical replications. Conflating technical and biological variability are not included. All data are collected from public domain.
Randomization	Randomization is not relevant to this study since each data was analyzed separately. The analyses reported in this paper do not involve between sample comparison.
Blinding	Blinding is not relevant since no data collection was involved in the present study.

Reporting for specific materials, systems and methods

We require information from authors about some types of materials, experimental systems and methods used in many studies. Here, indicate whether each material, system or method listed is relevant to your study. If you are not sure if a list item applies to your research, read the appropriate section before selecting a response.

Materials & experimental systems

n/a	Involved in the study
☒	☐ Antibodies
☒	☐ Eukaryotic cell lines
☒	☐ Palaeontology and archaeology
☒	☐ Animals and other organisms
☒	☐ Clinical data
☒	☐ Dual use research of concern
☒	☐ Plants

Methods

n/a	Involved in the study
☒	☐ ChIP-seq
☒	☐ Flow cytometry
☒	☐ MRI-based neuroimaging
